# Prevalence and antibiotic resistance profile of thermophilic *Campylobacter* spp. of slaughtered cattle and sheep in Shiraz, Iran

**Published:** 2016-09-15

**Authors:** Rahem Khoshbakht, Mohammad Tabatabaei, Saeid Hoseinzadeh, Mojtaba Raeisi, Hesamaddin Shirzad Aski, Enayat Berizi

**Affiliations:** 1 *Department of Food Hygiene, Faculty of Veterinary Medicine, Amol University of Special Modern Technologies, Amol, Iran; *; 2 *Department of Pathobiology, School of Veterinary Medicine, Shiraz University, Shiraz, Iran;*; 3 *Department of Food Hygiene, School of Veterinary Medicine, Shiraz University, Shiraz, Iran;*; 4 *Department of Public Health, School of Health, Golestan University of Medical Sciences, Gorgan, Iran.*

**Keywords:** Antibiotic resistance, Cattle, PCR, Sheep, Thermophilic *Campylobacter* spp.

## Abstract

Although poultry meat is considered as the main source for human *Campylobacter* infections, there is limited information about non-poultry sources. The present study was aimed to investigate the prevalence and the antibiotic resistance of thermophilic *Campylobacter *spp. in fecal samples of the cattle and sheep in Shiraz, Iran. A total of 302 fecal samples were obtained from clinically healthy, slaughtered cattle and sheep from Shiraz slaughterhouse. The animals were clinically healthy before being slaughtered. The samples were cultured according to the specific cultivation method under thermophilic conditions. The susceptibility of *Campylobacter *isolates were determined for 13 antimicrobial agents. All enriched samples and cultured isolates were targeted for polymerase chain reaction (PCR) detection of *16S rRNA* and multiplex PCR for determining their species. Among 302 fecal samples, 65 (21.5%) and 205 (67.8%) samples were positive for the presence of *Campylobacter* species with the cultivation and PCR techniques, respectively. All 65 distinct isolates were susceptible to neomycin and colistin and the isolates showed high resistance to cephalotin (83.0%) and ciprofloxacin (67.7%). After the multiplex PCR, 78.5% of total positive samples showed the simultaneous presence of *Campylobacter jejuni* and* Campylobacter coli*. In conclusion, the results emphasized that non-poultry farms are important as a possible source of *Campylobacter* infections.

## Introduction

 Campylobacter species, especially thermophilicCampylobacters like campylobacter jejuni and coli, are one of the important causes of diarrheal diseases in human. Campylobacter enteritis is the most frequently infection observed before the development of Guillain-Barré and Miller-Fisher syndromes, making the Campylobacter infection as a major public health issue.^1^These organisms widely discriminated in multitude of animal reservoirs showing varying degrees of resistance to different antibiotics.^2^^,^^3^ In Campylobacter enteritis, the macrolides and fluoroquinolones are considered the drugs of choice.^4^^,^^5^ However, in the past two decades, the antimicrobial resistance of Campylobacter spp. to the fluoroquinolones and macrolides has increased, mainly as a result of the approval of this group of antimicrobial for the use in food producing animals.^3^^,^^6^ Among the campylobacters, the thermophilic species particularly C. jejuni are the most frequently isolated bacteria from human infections.^5^ Whilepoultry meat is considered as the main source of human Campylobacter infection,there is growing evidence suggesting that the non-poultry sources can be equally important.^7^ Cattle, sheep and other food animals frequently carry C. jejuni and C. coli,^8^as commensals in their rumen and small intestine;^9^ and carcasses may be contaminated at slaughtering process by direct or indirect fecal contamination.^10^In this context, it is necessary to estimate the distribution and antimicrobial susceptibility of the bacteria associated with food animals. Currently, there is limited information on the prevalence of human pathogen Campylobacter spp. and their properties against antimicrobials in slaughtered cattle and sheep in Iran. The thermophilic campylobacters are important in diarrheal diseases in human and food animals can play a carrier role.^1^^,^^5^ the present study was conducted to determine the occurrence and antimicrobial resistance of thermophilic Campylobacter spp. isolated from the feces of slaughtered cattle and sheep in Shiraz, Iran. In addition, the identification of the microorganism using PCR method was compared with microbiological culture as a conventional strategy.

## Materials and Methods

**Sample collection and Campylobacter Culture.** From September 2011 to January 2013, a total of 302 fecal samples from cattle (n = 182) and sheep (n =120) were collected, at a slaughterhouse in Shiraz, Iran. The feces were taken from rectum of randomly chosen clinically healthy animals before slaughter, according to the method that was previously described.^11^ Briefly, fecal samples were collected in tryptic soy broth (TSB; Merck, Darmstadt, Germany) tubes and taken to the laboratory at 4 ˚C in less than 6 hr. For eliminating the other bacteria, 0.8 μM membrane filter (Sigma-Aldrich, Hamburg, Germany) was used and filtered samples were cultured in an enriched broth media, (TSB; 30 g L^-1^), dextrose (2.5 g L^-1^), sodium thioglycolate (0.5 g L^-1^), rifampicin (10 mg L^-1^), trimethoprim (10 mg L^-1^), vancomycin (10 mg L^-1^), ceftriaxone (10 mg L^-1^), amphotericin-B (10 mg L^-1^). Cultures then were incubated in a microaerophilic atmosphere (Anaerocult C; Merck, Whitehouse Station, USA) at 37 ˚C for 4 hr, followed by incubation at 42 ˚C for 44 hr. Thereafter, 50 μL of enriched samples in the TSB were cultured on selective agar, brucella agar base (41 g L^-1^) with 5.0% sheep blood and above antibiotics with identical dose.^11^ The preliminary identification of Campylobacter species was done according to the phenotypic characteristics; such as colony appearance, Gram staining, microscopic morphology, oxidase and catalase reactions.^6^ The strains C. jejuni (ATCC 33291) and C. coli (RTCC 2541) were included as positive controls in both culture and consequent PCR reactions. All above mentioned chemicals were obtained from HiMedia Laboratories Ltd. (Tarnaka, India) unless otherwise mentioned.

**Antimicrobial susceptibility test.** Susceptibility of Campylobacter isolates to 13 antibiotics were examined by the disk diffusion (Kirby Bauer’s) technique using Mueller-Hinton agar (Merck, Hamburg, Germany) supplemented with 5.0% de-fibrinated sheep blood, according to the Clinical and Laboratory Standards Institute (CLSI) guidelines.^12^ The antibiotic discs and their concentrations were cefotaxime (30 μg, Polfa Tarchomin, Warszawa, Poland), cephalotin (30 μg, Polfa Tarchomin), chloram-phenicol (30 μg, Bayer, Wuppertal, Germany), nalidixic acid (30 μg), erythromycin (15 μg), gentamicin (10 μg), neomycin (10 μg), tetracycline (30 μg), ampicillin (10 μg), ciprofloxacin (15 μg), enrofloxacin (5 μg), colistin (10 μg) and tylosin (30 μg). The susceptibility of the C. jejuni and C. coli to each antimicrobial agent was measured and the results were interpreted in accordance with interpretive criteria provided by CLSI.^12^

**DNA preparation and PCR assay.** Each enriched sample in the TSB was used for DNA extraction. Moreover, after preliminary identification of Campylobacter spp., each campylobacter colony on the selective agar was used for DNA extraction. Thebacterial DNA was extracted and purified by the procedure described by Sambrook et al. using phenol-chloroform and CTAB/NaCl technique.^13^ The purity and concentration of the DNA were estimated by spectro-photometry at 260 and 280 nm (Nanodrop 1000; Thermo Fisher Scientific, Waltham, USA). Simple and multiplex PCR reactions were done for identification of Campylobacter isolates at genus and species (C. jejuni and C. coli) level, respectively, using specific primers (Table 1). The PCR amplifications were performedin 25 µL final volume.The reaction mixtures consisted of 2.0 µL of the DNA template (50 ng), 2.5 µL 10X PCR buffer, 1.0 µL dNTPs (50 µM), 0.2 µL (1 U) Taq DNApolymerase, (CinnaGen, Tehran, Iran), 1.0 µL (25 pmol) of each forward and reverse primersfor simple and multiplex PCR reactions (Table 1). The volume of the reaction mixture was reached to 25.0 µL using distilled deionized water. The thermal cycler (MJ Mini, BioRad, Hercules, USA) was adjusted under the following conditions: Initial denaturation at 94 ˚C for 4 min, followed by 35 cycles of denaturation at 94 ˚C for 1 min, annealing (as shown in Table 1) for 1 min and extension at 72 ˚C for 1 min. Final extension was carried out at 72 ˚C for 5 min and the PCR products were remained in the thermal cycler at 4 ˚C until they were collected.

**Table 1:** Primers used in PCR reactions for identification of *Campylobacter* genus and species

**Name of primer**	**Sequence (5` to 3`)**	**Target gene**	**Annealing temperature**	**Product size (bp)**	**Reference**
**MapAF** **MapAR**	CTATTTTATTTTTGAGTGCTTGTG GCTTTATTTGCCATTTGTTTTATTA	***mapA*** (*C. jejuni*)	52 ˚C	589	14
**Coli F** **Coli R **	AATTGAAAATTGCTCCAACTATG TGATTTTATTATTTGTAGCAGCG	***ceuE*** (*C. coli*)	52 ˚C	462	14
**PLO6** **CAMPC5 **	GGTTAAGTCCCGCAACGAGCCGC GGCTGATCTACGATTACTAGCGAT	***16S rRNA*** (Genus)	50 ˚C	283	15

**Fig. 1:**
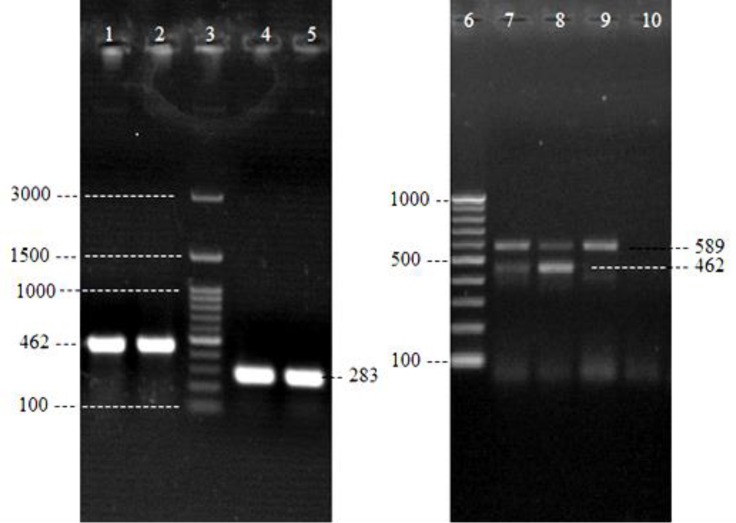
Agarose gel electrophoresis of *16S rRNA* genus specific (283 bp), *mapA* (589 bp) and *ceuE* (462 bp) gene, genus specific and multiplex PCR products, respectively. Lanes 1: Positive control for *ceuE* gene; 2: *ceuE *gene; 3: 100-bp plus DNA marker; 4: Positive control for *16S rRNA* genus specific; 5: *16S rRNA* PCR products of sample; 6: 100-bp DNA marker; 7 and 8: *mapA* and *ceuE* genes PCR products of samples; 9: Positive control for *mapA* gene; and 10: Negative control

Amplified products were separated by gel electro-phoresis on 1.5% agarose gel stained with ethidium bromide (0.5 µg mL^-1^, CinnaGen, Tehrran, Iran), and visualized in an ultraviolet light transluminator (BTS-20M; Uvitec, Cambridge, UK).The 100-bp DNA (Vivantis, Subang Jaya, Malaysia) and 100-bp plus DNA (CinnaGen) ladders were used as molecular size marker (Fig. 1).

**Statistical Analysis.** Data were analyzed using SPSS version 16.1 (SPSS Inc., Chicago, USA). Discrete variables were expressed as percentages and proportions were compared using the Chi-square test. Statistical significant difference was considered at value of p ≤ 0.05.

## Results

From a total number of302 fecal samples, 65 (21.5%) and 205 (67.8%) samples were positive for the presence of thermophilic Campylobacter species with cultivation and PCR procedures, respectively. When the cultivation method was compared with the PCR method, The PCR method had better specificity and sensitivity than cultivation methods with an overall agreement of 53.6%. Furthermore, a higher level of detection power was observed using the PCR method for the detection of campylobacter isolates. All samples with positive culture were also positive for the genus specific simple PCR. The PCR results showed that the prevalence of thermophilic Campylobacter in the cattle and sheep fecal samples were 130/182 (71.4%) and 75/120 (62.5%), respectively. Totally, from 205 PCR positive specimens, 161 (78.5%) samples showed positive results for both the C. jejuni and C. coli specific primers in the multiplex PCR reaction. In these PCR positive samples, 6 (2.9%) and 26 (12.6%) samples were positive for the C. coli and C. jejuni, respectively. Moreover, 12 (5.8%) samples were negative in the multiplex PCR, which were considered as other thermophilic Campylobacter species. The PCR method showed higher level of the specificity than the culture method. The multiplex PCR results showed the simultaneous presence of two thermophilic campylobacter species in positive samples, but the culture method could only detect one specie in each positive sample. The comprehensive results of distribution of thermophilic Campylobacter species among cattle and sheep fecal samples with culture and PCR methods are shown in Table 2.

**Table 2:** Prevalence of thermophilic *Campylobacter* species in cattle and sheep fecal samples. The data within the parentheses are presented as percentage

**Animal ** **source**	**Number of samples**	**Positive in culture method **	**Positive for ** ***16S rRNA*** ** PCR **	**Positive in multiplex PCR **			
				***C. coli***	***C. jejuni***	***C. coli *** **+ ** ***C. jejuni***	**Other spp.**
**Cattle**	182	42 (23.0)	130 (71.4)	3 (2.3)	16 (12.3)	104 (80.0)	7 (5.3)
**Sheep**	120	23 (19.1)	75 (62.5)	3 (4.0)	10 (13.3)	57 (76.0)	5 (15.0)
**Total**	302	65 (21.5)	205 (67.8)	6 (2.9)	26 (12.6)	161 (78.5)	12 (5.8)

Antibiotic susceptibility test showed high resistance to cephalotin (83.0%) and ciprofloxacin (67.7%) and low resistance to erythromycin (12.3%), neomycin and colistin (0.0%). Table 3 shows theresistance of the isolates to different antimicrobials. The results showed that C. coli was significantly more resistant than C. jejuni to nalidixic acid and erythromycin (p ≤ 0.05). In addition, the data showed that C. coli isolated from sheep were more susceptible than other isolates to these antibiotics.

**Table 3:** Antimicrobials resistance of *Campylobacter *isolates. The data within the parentheses are presented as percentage

**Antimicrobial agent**	**Cattle**			**Sheep**		**Total**
	***C. jejuni*** ** (n = 30)**	***C. coli*** ** (n = 12)**		***C. jejuni*** ** (n = 18)**	***C. coli*** ** (n = 5)**	
**Ampicillin**	13 (43.3)	3 (25.0)		9 (50.0)	2 (40.0)	27 (41.5)
**Chloramphenicol**	2 (6.6)	1 (8.3)		2 (11.1)	0 (100)	5 (7.6)
**Enrofloxacin**	6 (20.0)	3 (25.0)		2 (11.1)	0 (0.0)	11(16.9)
**Ciprofloxacin**	20 (66.6)	9 (75.0)		10 (55.5)	5 (100)	44 (67.6)
**Tetracycline**	9 (30.0)	2 (16.6)		4 (22.2)	0 (0.0)	15 (23.0)
**Gentamicin**	3 (10.0)	1 (8.3)		1 (5.5)	0 (0.0)	5 (7.6)
**Neomycin**	0 (0.0)	0 (0.0)		0 (0.0)	0 (0.0)	0 (0.0)
**Erythromycin**	2 (6.6)	3 (25.0)		1 (5.5)	2 (40.0)	8 (12.3)
**Nalidixic acid**	4 (13.3)	4 (33.3)		3 (16.6)	4(80.0)	15 (23.0)
**Colistin**	0 (0.0)	0 (0.0)		0 (0.0)	0 (0.0)	0 (0.0)
**Cephalotin**	27 (90.0)	10 (83.3)		17 (94.4)	0 (0.0)	54 (83.0)
**Cefotaxime**	7 (23.3)	2 (16.6)		6 (33.3)	2 (40.0)	17 (26.1)
**Tylosin**	6 (20.0)	2(16.6)		9 (50.0)	0 (0.0)	17 (26.1)

## Discussion

 Food animals have been incriminated as the main source for Campylobacter infection in humans.^3^^,^^16^The main source of carcass contamination is intestinal contents during manual skinning, evisceration, washing and processing in the slaughterhouse.^17^Therefore, determining its prevalence is the first step to assess the food safety continuum before setting targets and taking efficient measures to decrease animal pathogen carriage and finally reducing of the hazard of human infection.^18^ Most of the previous studies have investigated C. jejuni and C. coli in the diarrheic animals such as cattle and sheep,^19^^,^^20^ but studies related to healthy animals are limited.^2^^,^^18^^,^^21^The primary purpose of the present study was to investigate the prevalence of C. jejuni and C. coli in fecal samples of clinically healthy slaughtered sheep and cattle in Shiraz, Iran. The results of the present study showed 21.5% (65 of 302) of the examined animals were positive for Campylobacter spp. in routine cultivation method using the enrichment procedure and specific selective medium that was in accordance with other studies.^21^^,^^22^ The frequency of Campylobacter spp. among sheep isolates (19.1%) using culture method was in accordance with other studies conducted in Portugal,^23^ (15.0%), and Brazil,^24^ (20.0%) and did not significantly differ from the presence of the organism in cattle.Nevertheless, unlike the study of Kassa et al. the occurrence of Campylobacter spp. in cattle was higher than sheep by means of cultivation method in the present study.^16^ Other reports indicated the high prevalence of campylobacters in cattle.^18^^,^^25^^,^^26^These dissimilarities of the prevalence of the campylobacter among different animals may be due to the physiological differences of gastrointestinal tract or various flora and consistency of the feces of these animals.Conventional culture method for isolation of Campylobacter generally requires 4 days to show a negative result and 6 to 7 days to confirm a positive resultand this phenotypic distinction is not always accurate.^27^Faster identification of Campylo-bacter in feces would facilitate earlier implementation of proper strategies for treatment, control and prevention. In the present study, the occurrence of Campylobacter were 71.4% and 62.5% in the cattle and sheep fecal samples, respectively, using genus specific PCR; which indicates a high prevalence of campylobacters in these food animals. As a result, cultivation method does not supply a factual evaluation of the frequency of Campylobacter species in the sheep and cattle and other food animal samples. Furthermore, this method has lower sensitivity than PCR. The number of live microorganisms decreases during transport of the samples and many of the cells die and cannot show growth in cultivation methods. Accordingly, the true prevalence of Campylobacter in fecal samples obtained by PCR is indeed more than the prevalence obtained by culture. The PCR can show the presence of both dead and live cells in different samples. Multiplex PCR was carried out to determine the prevalence of C. jejuni and C. coli among genus specific PCR positive specimens and the isolates. Although other studies,^16^^,^^28^ reported the isolation of each species separately using the culture method (which was in agreement with the present study) surprisingly, in the present study, multiplex PCR results showed the simultaneous presence of two thermophilic species in 78.5% of Campylobacter positive samples. This fact confirms that these two species are in combination and cooperation in natural environment and their hosts’ milieu. Furthermore, the results showed that the specificity of the PCR method was better than conventional cultivation method. Totally, 12 specimens with positive PCR were negative in multiplex PCR, which were considered as other non-pathogen Campylobacter species.According to a previous study, C. coli has been found to be common in humans and chickens but rare in sheep and cattle,^25^however the present study showed high prevalence of this micro-organism con-currently with C. jejuni in healthy cattle and sheep fecal samples. This high prevalence may be due to the age of animals which are often slaughtered at the end of the breeding period as Besser et al. previously described the increase in prevalence of Campylobacter during breeding period.^29^

Nowadays, there is limited data on the antibiotic susceptibility patterns of the Campylobacter spp. isolated from various sources. Erythromycin and ciprofloxacin are two of the recommended antibiotics for treatment of the Campylobacter enteritis in human.^4^^,^^5^ According to the results, Campylobacter spp. isolated from cattle and sheep showed 12.3% resistance to erythromycin and high resistance to ciprofloxacin which can be a serious challenge for treatment of human campylobacteriosis associated with food animal origins. All 65 cultured isolates were susceptible to neomycin and colistin and showed low level of resistance to gentamicin and chloramphenicol. Gentamicin and chloramphenicol-resistant isolates were unusual and these results were similar to other studies.^2^^,^^3^^,^^6^ Some other studies showed high resistance to cipro-floxacin^2^^,^^6^ and erythromycin.^21^^,^^30^ However, the results of a few studies showed the susceptibility to erythromycin^2^^,^^3^ and ciprofloxacin.^3^^,^^31^ Comparing between C. jejuni and C. coli strains, the statistical analysis did not show significant difference in antibiotic resistance against the majority antimicrobials. However, these data showed that C. coli significantly (p ≤ 0.05)was more resistant than C. jejuni to nalidixic acid and erythromycin.

In conclusion, the results indicate the high prevalence of C. jejuni and C. coli, in healthy cattle and sheep as food animals, emphasizing the importance of non-poultry farms as possible sources of the Campylobacter infection. Resistance of C. jejuni and C. coli to the macrolides (e.g., erythromycin) and the fluoroquinolones (e.g., ciprofloxacin) was the most alarming finding in this study, which may be as a result of high consumption of these antibiotics in veterinary and human medicine. It seems that more control and prevention strategies are needed against thermophilic Campylobacter with animal origin. Moreover, we must have more vigilant usage of the antibiotics in food animals and establish a surveillance of developing resistance to antibiotics among animal isolates.
